# Enzyme response of activated sludge to a mixture of emerging contaminants in continuous exposure

**DOI:** 10.1371/journal.pone.0227267

**Published:** 2020-01-13

**Authors:** Georgiana Amariei, Karina Boltes, Roberto Rosal, Pedro Leton

**Affiliations:** Department of Chemical Engineering, University of Alcalá, Alcalá de Henares, Madrid, Spain; University of Siena, ITALY

## Abstract

The relevant information about the impacts caused by presence of emerging pollutants in mixtures on the ecological environment, especially on the more vulnerable compartments such as activated sludge (AS) is relatively limited. This study investigated the effect of ibuprofen (IBU) and triclosan (TCS), alone and in combination to the performance and enzymatic activity of AS bacterial community. The assays were carried out in a pilot AS reactor operating for two-weeks under continuous dosage of pollutants. The microbial activity was tracked by measuring oxygen uptake rate, esterase activity, oxidative stress and antioxidant enzyme activities. It was found that IBU and TCS had no acute toxic effects on reactor biomass concentration. TCS led to significant decrease of COD removal efficiency, which dropped from 90% to 35%. Continuous exposure to IBU, TCS and their mixtures increased the activities of glutathione s-transferase (GST) and esterase as a response to oxidative damage. A high increase in GST activity was associated with non-reversible toxic damage while peaks of esterase activity combined with moderate GST increase were attributed to an adaptive response.

## 1. Introduction

Pharmaceuticals and personal care products (PPCP) are one of the most relevant group of emerging pollutants because of their worldwide detection in practically all environmental compartments and their adverse biological effects [1–3]. PPCP includes many active substances, some of which, like antibiotics and antiseptics, specifically target bacteria and can also affect other microorganisms [4]. The extensive use and disposal of PPCP inevitably leads to their release to the environment either excreted in unmetabolized forms, or as active metabolites. Most PPCP reach the environment through industrial, hospital, and household wastewaters, which are discharged as effluents of wastewater treatment plants (WWTP) to receiving bodies [5–10]. WWTP represent the final defense for preventing PPCP from discharging into water environments, but the rate of biodegradation in conventional AS processes is low for most of these compounds [11]. AS processes are designed to remove chemical oxygen demand (COD), nutrient substances and pathogens, but not specifically to deal with emerging pollutants [11–13]. Conversely, the presence of substances with inhibitory or toxic effects to AS biological community may impair wastewater treatment performance [14].

Several studies examined the influence of pharmaceuticals on biological wastewater treatment processes, mainly focusing on the effects of pollutants on the efficiency of plants for their removal, microbial growth and the rate of COD and nutrients removal [15–18]. Arriaga et al. reported a decrease of COD removal in conventional AS reactors fed with pharmaceuticals [19]. Considerable research has been performed on the composition of functionality of microbial communities in AS upon exposure to anthropogenic chemicals [15, 16, 18–21]. Alvarino et al. studied the inhibitory effects of acetaminophen and doxycycline on the activity of nitrifying, denitrifying, and anammox biomass and found significant inactivation of ammonium oxidizing and denitrifying bacteria [13]. Zhang et al. studied the influence of environmentally relevant (1 μg L^-1^) concentrations of tetracycline on the microbial community of Sequent Batch Reactors and reported changes in microbial community and the proliferation of tetracycline-resistance genes [22]. Alterations in bacterial communities have also been found upon exposure to non-antibiotic polar pharmaceuticals [23]. In certain cases, it has been reported that continuous exposure to PPCP alters the composition of microbial community even in the absence of significant decrease in WWTP performance [24]. Some studies have addressed the impact of PPCP on the enzymatic activities of AS microbial consortia [25]. It has recently been found that an increase of the activity of oxidative stress enzymes could indicate low-to-moderate toxicity of non-steroidal anti-inflammatory drugs to AS communities [16]. However, there is a limited amount of work on this topic, and in particular addressing the impact of drug combinations to the enzymatic activity of AS. Therefore, should be paid more attention to the interaction between pollutants and AS [6].

In this work, we studied ibuprofen (IBU) and triclosan (TCS) as representative PPCP usually found in treated wastewaters [26, 27]. IBU is a widely used a non-steroidal anti-inflammatory drug, and one of the most used active pharmaceutical ingredients worldwide [28]. TCS is a broad-spectrum antimicrobial agent used as antiseptic, disinfectant and preservative in many consumer products including cosmetics, household cleaning products and materials such as medical devices, textiles and plastic ware [29, 30].

In a previous work, we demonstrated the toxic effects of IBU and TCS, alone and in combination to the microbial activity of AS in terms of viability and respiration for short-time exposure (1h) and non-acclimated sludge by using a batch experimental design [31]. Here, we evaluated the influence of IBU and TCS, alone and in combination to the microbial activity of AS in pilot AS reactors operating for 14-day upon continuous exposure. This study focused on: (1) the effects of IBU and TCS on the reactor performance parameters and (2) their influence on oxygen uptake rate, esterase activity and, for the first time, some key enzymes associated with oxidative stress namely glutathione S-transferase (GST) and catalase (CAT). This work provides new insights into the toxicity mechanisms of mixed emerging pollutants to a heterogeneous AS microbial community under continuous exposure.

## 2. Materials and methods

### 2.1. Chemicals

Triclosan, (TCS, C_12_H_7_Cl_3_O_2_) and ibuprofen (IBU, C_13_H_18_O_2_) both > 97% purity, were obtained from Sigma-Aldrich. The stock solutions of TCS and IBU were prepared in methanol and water, respectively and stored at 4 °C. Methanol in the testing solution was kept below 0.01% (v/v). Ultrapure water was generated from a Direct-Q^™^ 5 Ultrapure Water Systems from Millipore (Bedford, MA, USA) with a specific resistance of 18.2 MΩ cm at 25 °C. Monochlorobimane (MCB), 2',7'-dichlorofluorescin diacetate (H_2_DCF-DA), reduced glutathione (GSH) and dimethyl sulfoxide (DMSO, 99.9%) were purchased from Sigma-Aldrich. The components of synthetic feed were acquired from Conda-Pronadisa (Spain).

### 2.2. Experimental setup and procedure

The inoculated sludge was obtained from a local municipal WWTP (Seville, Spain) with a capacity to process 255,000 m^3^ of raw domestic wastewater per day and operating at SRTs of 2.54 days, and maintained in a bubble column reactor as described in Amariei et al [31]. The AS was fed using a Synthetic Sewage Feed, based on OCDE 209 standard medium with the following composition: peptone 16.0 g L^-1^, beef extract 11.0 g L^-1^, urea 3.0 g L^-1^, NaCl 0.7 g L^-1^, CaC_12_·2H_2_O 0.4 g L^-1^, MgSO_4_·7H_2_O 0.2 g L^-1^, and K_2_HPO_4_ 2.8 g L^-1^ [32].

**Fig 1:**
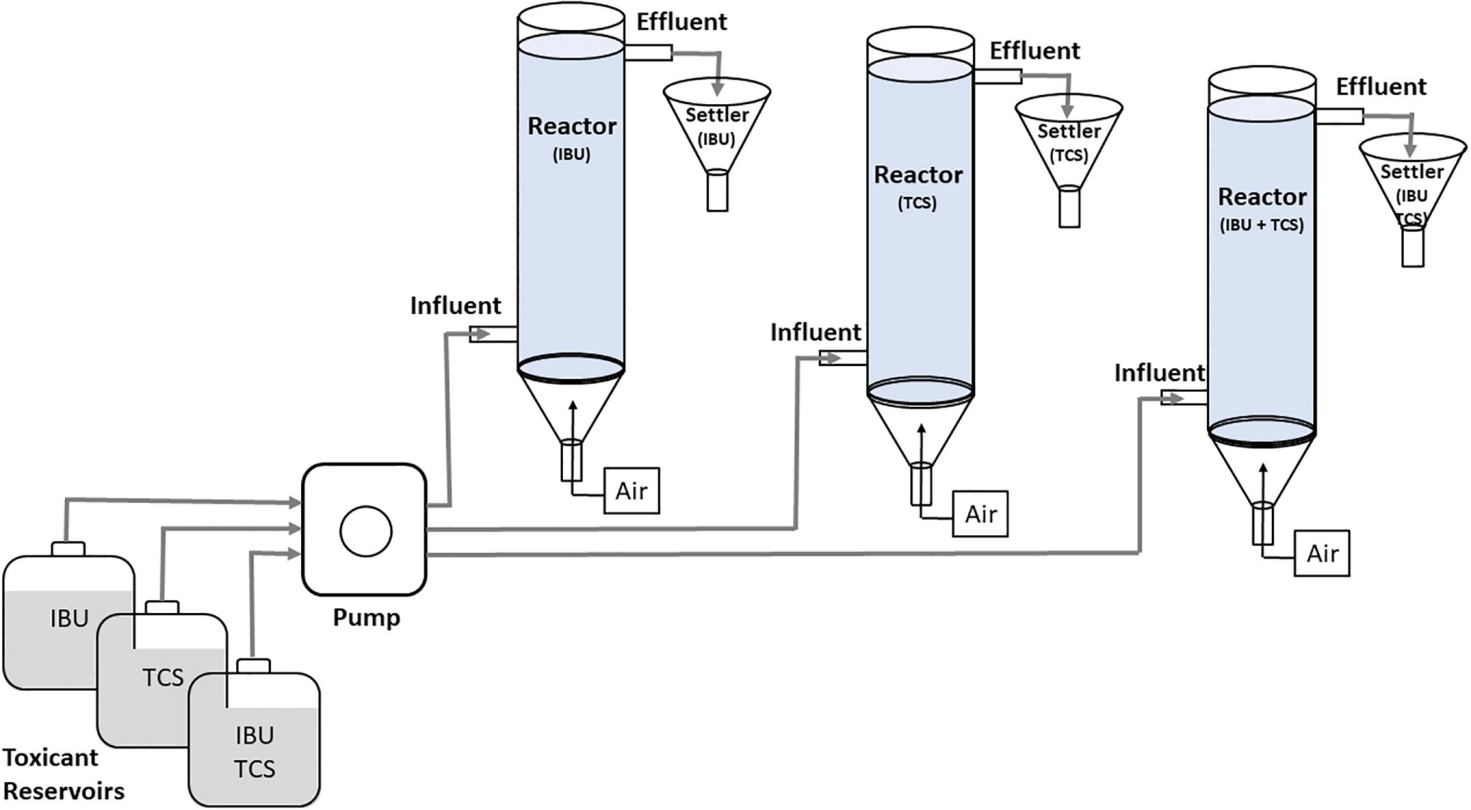
Diagram of the activated sludge laboratory systems.

The lab-scale AS systems used in this work were formed by three continuous aerated reactors and settlers, operated in parallel (Fig 1). The reactors were glass columns 41 cm height, 7.5 cm diameter and 2 L working volume. The air was supplied through a fine bubble diffuser set at the bottom of every reactor. For the startup of reactor operation, 300 mL of sludge (3 g/L TSS) taken from the settler of the bubble column reactor were added to every reactor as inoculum. The synthetic influent was continuous feed to the reactors at fixed rate by a peristaltic pump. The hydraulic retention time under operating conditions was 24 h. 50% of biomass from the settler was returned to the reactor in order to maintain the desired SRT and biomass concentration. The reactors were operated at food-to-microorganisms (F/M) ratios of 0.72 ± 0.084 g COD/g biomass at the starting of exposure experiments in order to avoid possible negative effects on the bacteria due to the limitation of food under toxic exposure experiments. Dissolved oxygen (DO) concentration was maintained at 3 mg L^-1^, temperature at 20 ± 2 °C and pH at 8.0 ± 0.1, close to the pK_a_ of TCS reported to be 8.1 [33]. Prior to exposure experiments, the reactors were operated continuously for 120 days under pseudo steady-state [34]. Acclimation was considered successful after this time as no significant changes in operating parameters (oxygen concentration, pH and biomass concentration) were observed. The biomass concentration, expressed as total suspended solids (TSS), was in the 300–500 mg L^-1^ range throughout all the experiments with minor deviations. The stability of pollutants under continuous bioassay conditions was verified by high pressure liquid chromatography (HPLC) before initiating the exposure experiments.

For the exposure study, the bioreactors operated in parallel with individual pollutants and their mixtures. The bioreactors were monitored for 14 days, divided into two periods. The first 7 days they operated with continuous dosing of IBU (2 mg L^-1^) and TCS (0.05 mg L^-1^) as well as their mixture (2 mg L^-1^ IBU + 0.05 mg L^-1^ TCS) in the influent. During the second period, starting day 8th, the dosing of pollutants doubled: 4 mg L^-1^ IBU, 0.10 mg L^-1^ TCS and for their mixture 4 mg L^-1^ IBU + 0.10 mg L^-1^ TCS. The amount of pollutants fed with the influent are listed in Table 1 expressed in mg day^-1^. Relative to the dry matter in AS, the pollutants were fed at rates of 125 mg kg^-1^ and 5000 mg kg^-1^ for TCS and IBU respectively during Period I and twice these figures for Period II. These figures were chosen to approach the upper estimates reported for the mass of PPCPs adsorbed or in general accumulated in the sewage sludge, which are 133 mg kg^-1^ and 2988 mg kg^-1^ for TCS and IBU respectively [28]. The concentrations in the influent, in the milligram per liter range, are representative of the whole amount of pharmaceuticals and personal care products reaching usual WWTP. For example, a one-year monitoring study quantifying 50 individual pollutants in a WWTP receiving urban wastewater over, which included PPCA and some metabolites found average and maximum global concentrations of 0.12 and 0.32 mg/L respectively [27]. The operational stability of reactors was carefully assessed before starting the runs.

**Table 1:** Mean amounts of ibuprofen (IBU) and triclosan (TCS) removed and standard deviations (n = 3) in the influent and effluent reactors loaded with pollutants alone or in binary mixture. The amount removed refers to the liquid phase. All amounts are expressed in mg day^-1^.

**IBU in mg/day**									
		IBU				IBU+TCS			
	Time (h)	Influent	Effluent		Removed	Influent	Effluent		Removed
			Liquid	Sludge			Liquid	Sludge	
**Period I**	24	6.60	3.60±0.27	0.07±0.01	2.93±0.28	6.60	2.20±0.17	0.01±0.01	4.39±0.17
	48		3.38±0.22	0.24±0.02	2.98±0.23		2.68±0.20	0.01±0.01	3.91±0.20
	96		2.93±0.25	0.13±0.01	3.54±0.27		2.09±0.16	0.15±0.01	4.36±0.17
	168		2.90±0.22	0.07±0.01	3.64±0.22		1.44±0.10	0.18±0.02	4.98±0.12
**Period II**	24	13.20	1.28±0.10	0.11±0.01	11.81±0.10	13.20	9.03±0.57	0.33±0.03	3.84±0.71
	48		1.15±0.09	0.47±0.04	11.58±0.12		7.20±0.36	0.57±0.05	5.43±0.59
	96		1.69±0.05	0.32±0.02	11.19±0.06		4.82±0.54	0.32±0.03	8.06±0.39
	168		0.64±0.13	0.16±0.02	12.40±0.15		3.71±0.28	0.12±0.01	9.37±0.29
**TCS in mg/day**									
		TCS				IBU+TCS			
	Time(h)	Influent	Effluent		Removed	Influent	Effluent		Removed
			Liquid	Sludge			Liquid	Sludge	
**Period I**	24	1.65	0.03±0.01	0.01±0.01	1.61±0.01	1.65	0.06±0.01	0.07±0.00	1.52±0.01
	48		0.04±0.01	0.11±0.01	1.50±0.01		0.06±0.01	0.23±0.02	1.36±0.03
	96		0.04±0.02	0.02±0.01	1.59±0.02		0.05±0.01	0.15±0.01	1.45±0.02
	168		0.06±0.02	0.01±0.01	1.58±0.02		0.05±0.01	0.07±0.01	1.53±0.01
**Period II**	24	3.30	0.05±0.01	0.02±0.01	3.23±0.01	3.30	0.03±0.01	0.04±0.01	3.22±0.05
	48		0.05±0.01	0.02±0.01	3.23±0.01		0.03±0.01	0.04±0.01	3.23±0.05
	96		0.07±0.01	0.02±0.01	3.21±0.01		0.03±0.02	0.04±0.01	3.22±0.05
	168		0.05±0.02	0.01±0.01	3.24±0.02		0.03±0.01	0.04±0.02	3.23±0.05

Samples of sludge and reactor effluent were withdrawn for analyses at prescribed times during the operation. Analyses of IBU and TCS concentration, COD (chemical oxygen demand), TOC, and ammonia-, nitrite-, and nitrate nitrogen were performed from the samples collected from the influent, effluent and sludge, as described in section 2.4. All assays were carried out in triplicate and the results were expressed as mean plus/minus standard deviation.

### 2.3. Microbial activity

The microbial activity was assayed by studying the oxygen uptake rate, esterase activity, oxidative stress and antioxidant enzyme activities. All measurements were carried out in triplicate and the results were expressed as mean standard deviation.

#### 2.3.1. Oxygen uptake rate

The specific oxygen uptake rate (SOUR) was determined as a measure of the whole aerobic biomass activity using the methodology developed elsewhere [31]. Briefly, the respirometry assay was carried out using a Oxygraph Hansatech System (Germany), consisting of a S1 Clark Type polarographic oxygen electrode in an enclosed cell equipped with magnetic stirring. The cell was filled with 2 mL of each sample and the changes in the dissolved oxygen (DO) were monitored. The values are expressed as grams of oxygen consumed per gram of biomass per day (day^-1^).

#### 2.3.2. Esterase activity

The fluorescein diacetate (FDA) hydrolysis technique was applied for evaluation of total esterase activity. FDA is a non-fluorescent molecule that diffuse into cells and are hydrolysed by intracellular non-specific esterases of bacteria [35]. For this, 195 μL samples of reactor were analyzed in 96-well microplates by adding 5 μL of FDA (0.02% w/w in DMSO) to each well. Fluorescence was measured using a fluorometer (ThermoScientific^™^ FL, Ascent) at excitation and emission wavelengths of 485 and 520 nm, respectively. The incubation time for staining was 30 min at 25 °C.

#### 2.3.3. Reactive oxygen species (ROS) and antioxidant enzyme activity

The intracellular ROS in the sludge cells was measured by means of the H_2_DCF-DA assay method according to the procedure reported in the literature [36]. Prior to the assay, the sludge liquor was first centrifuged at 10000 x g relative centrifugal force for 10 min and washed with 0.85% (w/w) NaCl solution. Then, the collected pellets were re-suspended in 0.85% (w/w) NaCl solution and incubated with 20 μl mol L^-1^ H_2_DCF-DA for 30 min. The mixed liquor was transferred into 96-well microtiter plates (ThermoScientific^™^ FL, Ascent) for fluorescence spectroscopy at excitation and emission wavelengths of 495 and 525 nm, respectively.

The enzyme-containing fractions from AS samples were obtained by sonication method. In brief, ice cooled samples of 10 mL sludge (~ 0.5 g TSS L^-1^) were individually homogenized using an ultrasonic cell disintegrator Sonics-VibraCell (BioBlock Scientific, France) with a power density of 1 W/mL for 5 (net) min in intervals of 5 s with 5 s breaks to avoid sample heating [37]. The crude cell lysate was subsequently purified through 0.45 μm and 0.20 μm syringe filters (Whatman 25 mm GD/X polyethersulfone membrane with glass microfiber prefilter). GST and CAT activities of the resulting cell lysates were assayed immediately after extraction [33].

GST activity was assessed using MCB as substrate and following the method of Nauen and Stumpf with minor variations [38]. In brief, the total reaction volume in each well was 150 μL, consisting of 25 μL sample aliquots, 25 μL potassium phosphate buffer (100 mM, pH7), 50 μL MCB (1% v/v ethanol), and GSH (3 mM). After 20 min of incubation at 22 °C, the GSH–bimane adduct was determined at 465 nm, upon exciting at 390 nm using a ThermoScientific^™^ FL, Ascent microplate reader.

The method for determining the activity of CAT enzyme was based on the O_2_ production caused by H_2_O_2_ reduction in presence of CAT enzyme [39]. The Oxygraph Hansatech System with a Clark-type oxygen electrode described before was used for this purpose following a method described elsewhere [31]. Briefly, the test cell was filled with 1.9 mL of phosphate buffer (pH 7, 50 mM), 100 μL enzyme extracts and 1mL H_2_O_2_ (0.003%). The changes in DO due to H_2_O_2_ reduction were monitored and recorded. The results were expressed as O_2_ produced/mL of bacterial biomass.

#### 2.3.4. Live/Dead distribution on AS

Cell integrity and metabolic changes of microbial consortia in AS during pollutants exposure were also visually examined using confocal laser scanning microscopy (CLSM). Live/Dead BacLight Bacterial Viability kit (Molecular Probes, Invitrogen Detection Technologies, Carlsbad, CA, USA) was used to evaluate bacterial viability in the sludge. In brief, this method differentiates viable and no-viability cells using Syto9, a fluorescent nucleic acid stain capable to penetrate cell membrane and bind DNA, and propidium iodide (PI), which is a fluorescent stain marking only membrane-damaged non-viable cells. The excitation/emission were 480/500 nm for Syto9 and 490/635 nm for PI. The micrographs were obtained in a Leica Microsystems Confocal SP5 fluorescence microscope. For this study, sludge samples (1 mL) were taken from the reactors before starting the exposure experiment: 0 days (Control), and at 7 days (Period I), and 14 days (Period II), in order to observe the effects of pollutants at the beginning and end of each period of exposure, respectively.

## 2.4. Analytical methods

COD was measured using spectrophotometer NOVA60 (Merck) after 2 h digestion. TOC values were determined by TOC-VCSH Total Organic Analyzer, Shimadzu. For the analysis of IBU and TCS, a solid phase extraction (SPE) was performed using, by 30 mg/3 mL Strata-X 33u Polymeric Reversed Phase cartridges (Phenomenex) as pre-concentration technique prior to quantitative determination. The extraction protocol was similar to that described elsewhere [40]. IBU and TCS were quantified by HPLC using an Agilent LC 1260 system, equipped with a 1260 Quaternary pump VL, 1260 DAD detector and automatic 1260 ALS injector. The column used was a Promosil C18 4.6 x 150 mm column. In each analysis, 50 μL samples were injected using a mobile phase consisting of 70% acetonitrile and 30% acid water (at pH 2) at a flow rate of 1 mL/min [31]. A pH-meter and an oximeter (Crison) were used to determine the pH and DO.

## 2.5. Statistical analysis

A two-way ANOVA coupled with Tukey’s HSD (honestly significant difference) post-hoc test was performed for comparison of means. Statistically significant differences were considered to exist when p-value < 0.05. Results were provided as average and standard deviation.

## 3. Results and discussion

### 3.1. Bioreactor performance

Table 1 shows the concentration of IBU and TCS in reactor influent and effluent for the three operational conditions used in this work (IBU, TCS and IBU+TCS) together with the amount removed from the aqueous phase (liquid) of effluent expressed in mg day^-1^. In all cases, most of the remaining IBU and TCS were dissolved in the effluent with a minimum part adsorbed on sludge. The amount removed during the Period I represented 50 ± 6% for IBU and 95 ± 3% for TCS. During Period II, after 7 days of acclimation, the removal of IBU increased to 89 ± 4%. TCS also increased slightly to 98 ± 1%.

In reactors treating binary mixtures of IBU and TCS, the removal efficiencies were not very different from reactors fed with IBU or TCS alone. The average removal for IBU during period I was 67 ± 7%, even higher than when IBU was fed alone. At the beginning of Period II the removal efficiency for IBU dropped until 20–30% in reactor operating with IBU+TCS mixture that in part recovered thereafter. For TCS, the removal was 89 ± 5% and 98 ± 1% (IBU+TCS reactors), only slightly lower than in reactors treating TCS alone. High removal efficiencies of anti-inflammatory drugs in aerobic treatment processes and low sorption potential of IBU on sludge have already been reported elsewhere [16]. In our case, removal efficiencies for TCS were mostly > 90%, both when fed alone or in combination with IBU. High removal efficiency of TCS has been reported elsewhere and attributed to biologically degradation [41]. The part related to the PPCPs absorbed on the components of the AS system (such as reactors, tubing etc.) was insignificant.

**Fig 2:**
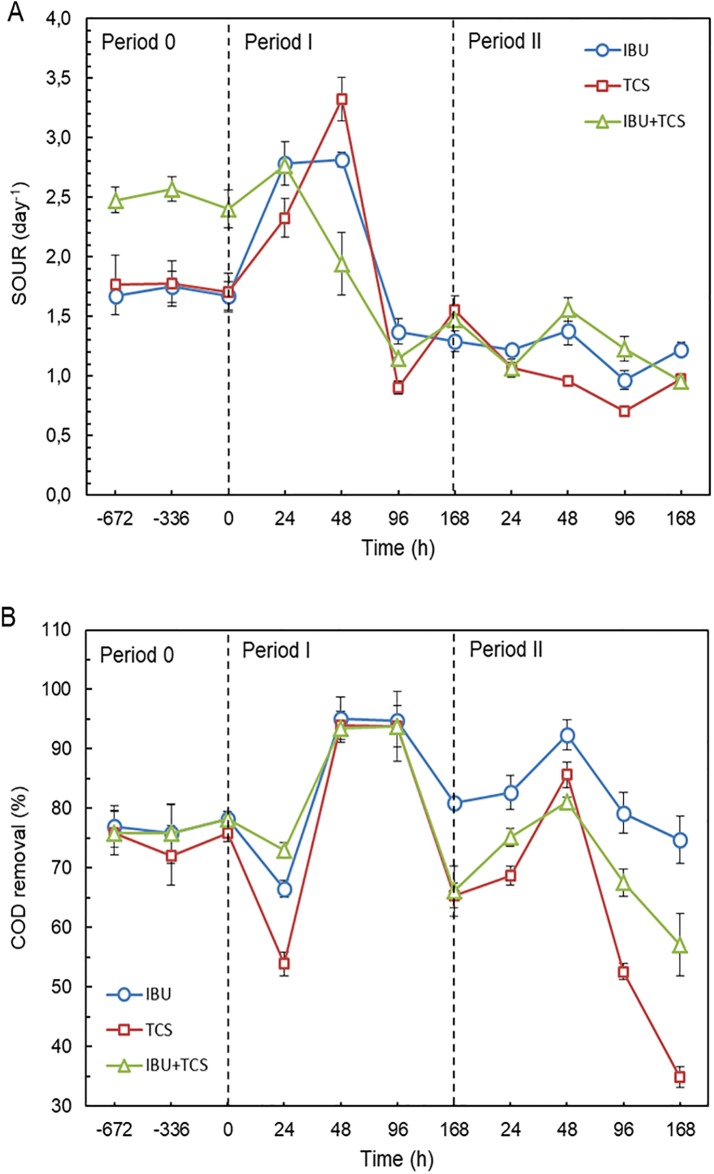
Effect of ibuprofen (IBU), triclosan (TCS) and their mixture on oxygen uptake (expressed as SOUR in day^-1^) and chemical oxygen demand (COD) removal during 14-day assays. (Period 0 represents the stable operation prior to the introduction of toxics).

The overall performance of bioreactors is shown in Fig 2. Fig 2A represents the evolution of SOUR upon continuous introduction of IBU, TCS and IBU+TCS during Period I (7 days with IBU 2 mg L^-1^, TCS 0.05 mg L^-1^ or 2 mg L^-1^ IBU + 0.05 mg L^-1^ TCS) and Period II (7 days with IBU 4 mg L^-1^, TCS 0.10 mg L^-1^ or 4 mg L^-1^ IBU + 0.10 mg L^-1^ TCS). The results showed a clear stimulation during the first 24–48 h after the introduction of pollutants, with maximum SOUR values even doubling the stationary state before exposure (~ 2 day^-1^). A peak in SOUR was not observed when doubling concentrations (Period II). During this period SOUR tended to decrease upon toxicant exposure. Our results showed that COD removal rate in acclimated reactors was in the 75–78% range (Fig 2B). During exposure experiments, the average COD removal showed a pattern similar to SOUR with COD removal increasing ~ 20% after 48 h and a subsequent decrease. During period II, with higher concentration of pollutants, COD removal also increased (48 h, 15–30%) in all reactors. COD removal eventually decreased at the end of Period II.

In the current study the amount of IBU and TCS used for Period I were 125 mg kg^-1^ and 5000 mg kg^-1^ respectively (twice during Period II) representing 1% and 5% respectively of their individual short-term EC_50_ values. A previous study performed according to the Standard Guideline OECD Test Guide 209 showed inhibitory effect of IBU and TCS to the respiration activity of AS measured in short-term (60 min) exposure [32]. The short-term EC_50_ value for TCS was 0.32 ± 0.07 mg L^-1^ for a concentration of AS of 125 mg L^−1^, which represented 2560 mg kg sludge^-1^. The short-term EC_50_ for IBU was of 64 ± 13 mg L^−1^ or 512000 mg kg sludge-1 for the same conditions [31]. The continuous introduction intended to mimic a realistic scenario and to avoid the artifacts derived to the lack of acclimation of sludge. Figure A in S1 File, shows the evolution of dissolved oxygen, pH and biomass concentration during the runs. Dissolved oxygen was stable along the assays, pH presented a continuous and moderate increase from 7.26 to 8.14 and the concentration of biomass was essentially constant at about 400 mg/L with a slightly decrease during the first 24–48 h of Period I. The overall operational parameters were consistent with the assumption that the acclimation of microorganisms created a stable state for microbial biomass [42].

The effect of TCS to the microbial community function in anaerobic digesters has been described by McNamara et al. who found increased methane production and transient divergence of community structure in reactors treating relatively low (5–50 mg/kg) TCS concentration [4]. TCS was shown to alter community structure by selecting resistant microorganisms [30]. The stimulatory effect of low (0.05–1.0 mg L^-1^) TCS concentration upon sludge SOUR was observed before and attributed to increased microbial metabolic rates linked with uncoupling of oxidative phosphorylation [43]. An initial stimulation followed by a decreased at the end of the two one-week period was also observed in this work for COD removal, suggesting a balance between acclimation and toxicity.

The stimulation of microbial activity upon stress conditions has been observed in different stress circumstances. For example, increased salinity generally inhibits the bioactivity of sludge microorganisms, but the selective growth of microbes with higher tolerance to saline environment may lead to an increase in microbial respiration rates [44]. Sorption, volatilization, photodegradation, and microbial degradation are important routes that eliminate PPCP from AS systems. However, several contradictory bio- and photo-transformation results were reported, while some studies suggest that the PPCP fraction removed by volatilization could be neglected due to low Henry coefficient values [45]. Different microbial processes may be involved such as the induction of different enzymatic pathways or the metabolization of different intermediates as an adaptation mechanism upon exposure to new chemicals, which may explain the observed changes in removal efficiencies and operational parameters [46]. A sudden stress given by a step increase in the influent concentration of toxicants has been shown elsewhere to be followed by a physiological adaptation period [47].

It is important to determine whether the impact of changes in toxicant concentrations on AS is reversible or not. Our results showed a possible warning situation in Period II (with higher concentration of IBU and TCS), at the beginning of which a sharp decrease in the removal of IBU was observed in reactors treating IBU+TCS mixture. At the end of Period II a clear decrease in COD removal was probably indicating a non-recoverable situation. Generally, the operators of WWTP take into account the quality of the effluent and the physical characteristics of the sludge to take measures on operational parameters. In the following section of this article we describe the results of a series of enzymatic activity tests in an attempt to provide a further insight into the mechanisms behind the stress conditions associated to emerging pollutants and their relationship with AS performance.

### 3.2. Enzymatic activity in AS

**Fig 3:**
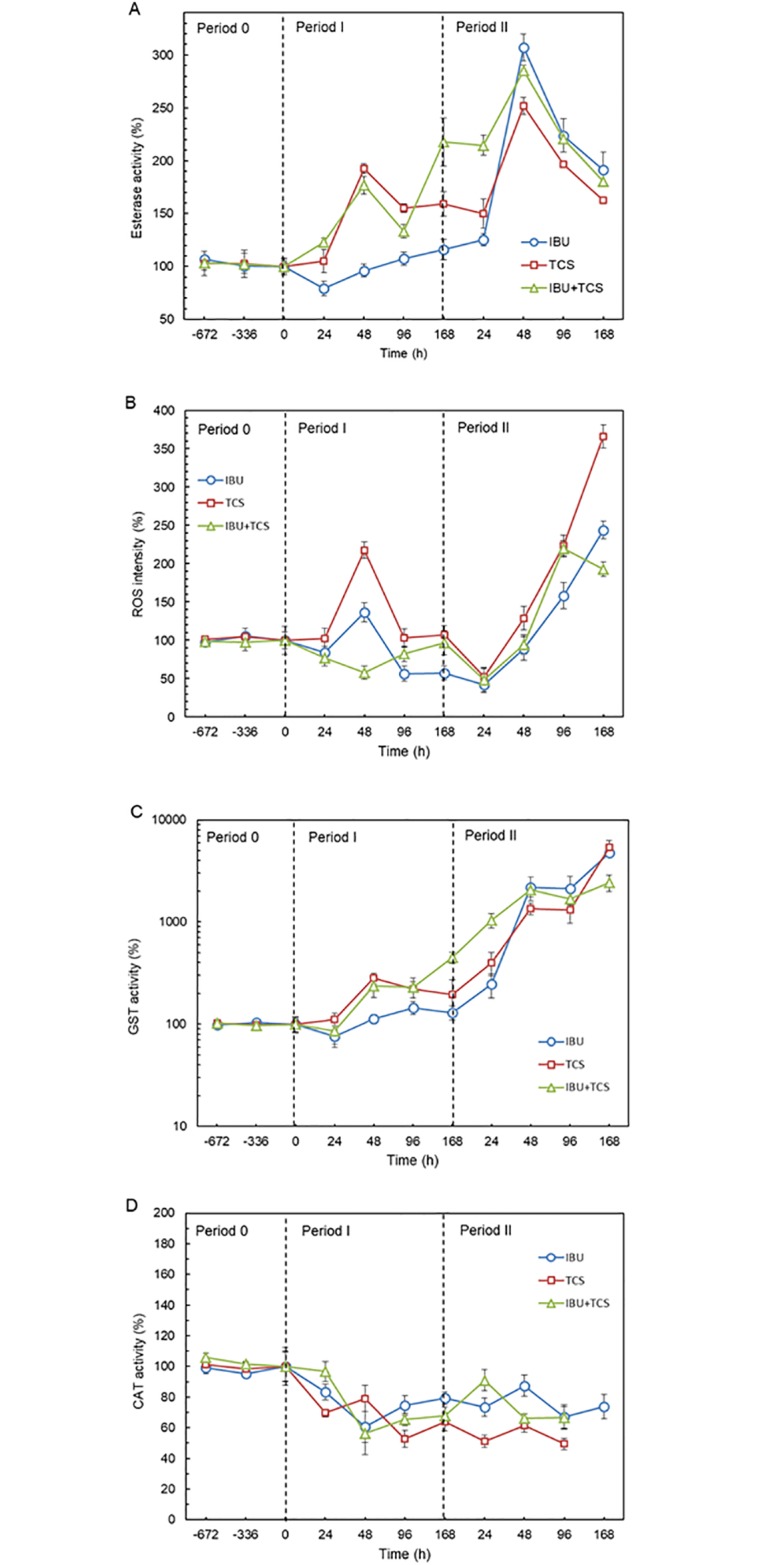
Effect of ibuprofen (IBU), triclosan (TCS) and IBU+TCS mixture on oxidative stress, ROS (A), and esterase (B), glutathione s-transferase, GST (C) and catalase, CAT (D) activities during 14-day assays.

The effect of IBU, TCS and IBU+TCS to the enzymatic activity of AS is shown in Fig 3A to 3D. Fig 3A displays the evolution of the average esterase activity expressed as percentage of the stable value recorded before introducing pollutants with the bioreactor feed. The results showed that IBU slightly inhibited esterase activity after the first 24 h (< 15%) followed by a moderate and continuous increase during the rest of Period I. TCS and IBU+TCS mixture induced higher esterase activity (more than doubling controls) and even higher values were recorded after 48 h during Period II to decline thereafter during the last part of the runs. The comparison with literature data is difficult due to the scarcity of toxicological studies dealing with specific emerging pollutants to the metabolic activity of acclimated AS communities. One previous study using short-term (60 min) exposure and non-acclimated sludge, showed EC_50_ values for the inhibition of esterase activity in activated exposed to IBU and TCS of 633 ± 63 mg L^−1^ sludge (125 mg L^−1^ of AS) and 1.94–5.34 mg L^−1^ (125–500 mg L^−1^ of AS) respectively [31]. The effect recorded here showed that low concentration and long-term exposure did not cause a decrease of the esterase activity. Conversely, we observed a stimulatory response in line with SOUR and COD as indicated before. Esterase activity in AS is driven by diverse esterase enzymes with broad, and partially overlapping, substrate specificity, which has been proposed to characterize the biological activity in wastewater treatment plants [48]. Esterase activity is a measure of microbiological activity and viability, but it has also been associated with stress responses. An increase in esterase activity has been described as physiological acclimation to stringent nutrient limitation in AS communities [49]. The operational conditions of bioreactors, including substrate, pH and temperature, also affect esterase activity. In our case, the pH of the reactors presented a continuous and moderate increase from 7.26 to 8.14 during the assay (Figure B S1 File), which may partially explain the general trend to esterase activity increase. Accordingly, it has been shown that esterase enzymes are most active at pH about 8.5, with less activity at pH ≤ 5.5 and deactivation at pH ≥ 8.5 [50]. The activity peaks observed in 48 h samples, are probably due to an adaptive response.

Typically, ROS are formed in cells through the reduction of oxygen by biological reducing agents such as NADH and NADPH, with the assistance of electron-transfer enzymes or through redox-active chemical species such as quinones and transition metals. Oxidative stress is a key cellular response in organisms exposed to environmental pollutants that takes place when the generation of ROS exceeds the capacity of antioxidant defenses. Low concentrations of ROS may facilitate signal transduction, enzyme activation and other cellular functions, but high concentration of ROS damages DNA, proteins or lipids and can lead to cellular transformations [51, 52]. In the present study, we observed a significant increase in ROS levels with respect to controls in all cases. The results (Fig 3B) show a transient increase of ROS levels during the exposure to the lower concentrations of IBU and TCS (Period I) that turned into a sharp (over three-fold) increase during Period II. The mixture IBU+TCS induced lower ROS levels than IBU and TCS alone, which is noteworthy considering the mixture uses jointly the same concentration of both pollutants when dosed alone. Organic pollutants have been linked to ROS overproduction, but to the best of our knowledge, there is no report on the capacity of ibuprofen and triclosan to induce significant ROS overproduction in AS.

Glutathione transferases are a group of eukaryotic and prokaryotic antioxidant enzymes that prevent the cellular damage caused by metabolically- and environmentally-produced ROS. GST transforms the reduced glutathione (GSH) used as a direct ROS scavenger to a variety of electrophilic compounds in a detoxification pathway. GST plays a crucial role in cell metabolism by protecting against oxidative damage and, therefore, GST has been used widely as a biomarker for assessing the toxic effects of pollutants that generate oxidative stress and moderate GST activity increase has been linked to detoxification routes upon pollutant exposure in bacteria [53]. The effects of TCS and IBU, individually and in combination, on GST activity are shown in Fig 2C. GST activity was raised by TCS, IBU and TCS+IBU after the first 24 h of exposure with an exponential increase to very high values during Period II. Our results demonstrated that the damage associated to IBU and TCS trigger a strong GST activity in AS microorganisms. The dramatic increase in GST activity upon exposure to the higher concentration of pollutants is parallel to the ROS increase observed for the same conditions (Fig 3B).

The increase in GST and esterase activities was linked to the oxidative damage produced to AS microorganisms. A continuous increase in GST activity would be the fingerprint of a non-reversible damage that compromised bacterial viability and led to microbial decline and loss of bioreactor performance. Peaks of esterase activity combined with moderate GST increase would be associated to adaptive responses.

Finally, Fig 3D shows CAT activity profile, which decreased during the first 48 h of Period I to stabilize thereafter irrespective of the higher pollutant concentration used during Period II. CAT is an antioxidant enzyme that indirectly takes part in the contaminant metabolization by targeting hydrogen peroxide. When ROS exceeds the scavenging capacity of superoxide dismutase and CAT, they become CAT inhibitors [54]. An enhancement of CAT activity has also been reported during the first 24 h after pollutant-induced oxidative stress, that recover thereafter [55]. Our data showed that the continuous exposure to 0.05 mg L^-1^ TCS and/or 2 mg L^-1^ IBU decreased CAT activity, which was probably the consequence of a damage in the antioxidant defense system that did not revert after acclimation.

Moreover, there were significant differences (p < 0.05) between the concentrations and the control as well as the time exposure for the end-points assayed in this work. The differences between the concentrations in ROS and CAT measurements for IBU and TCS were not significant (p > 0.05).

**Fig 4:**
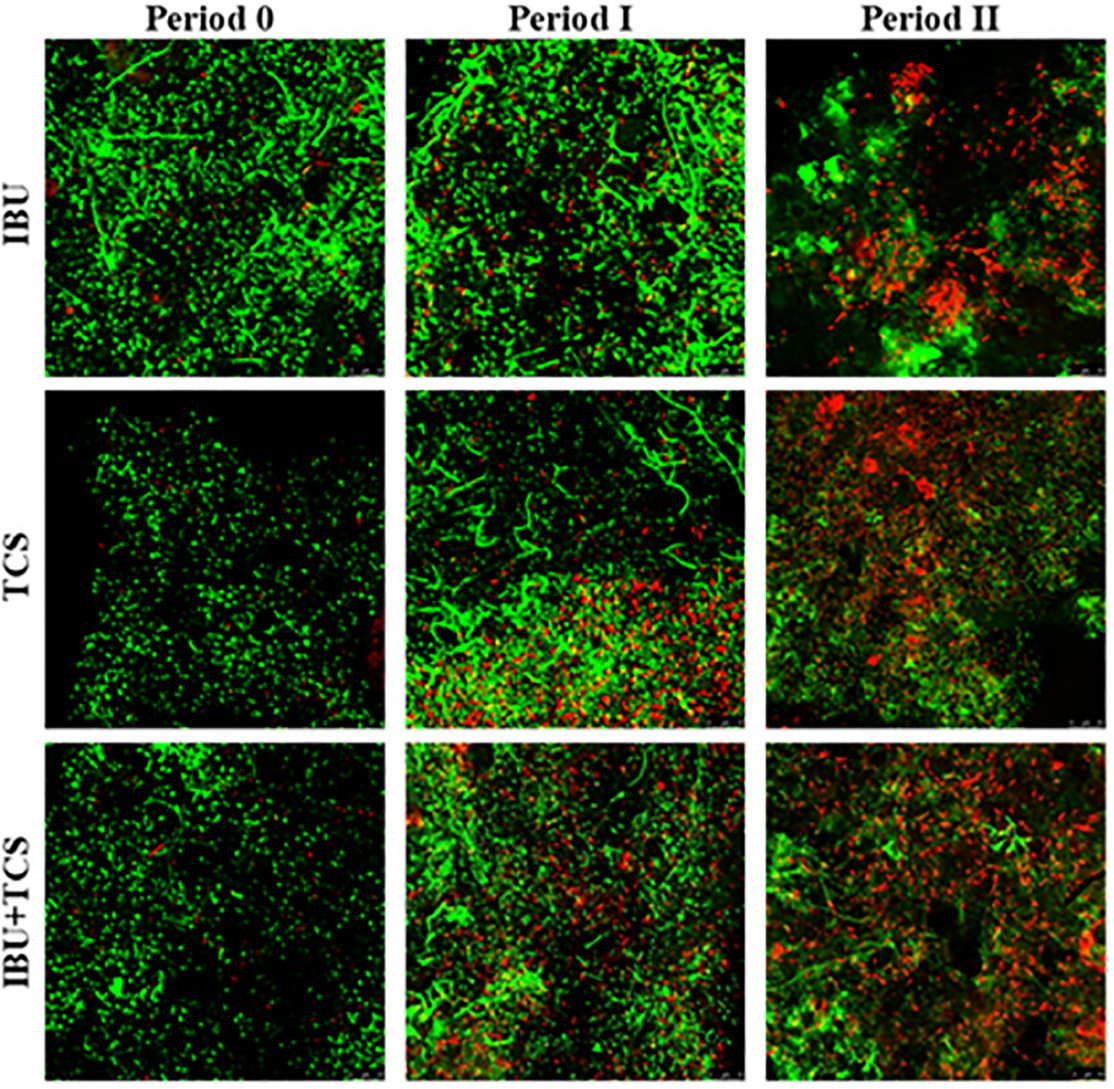
Live/Dead confocal micrographs (scale bar 10 μm) of activated sludge exposed to individual pharmaceuticals (ibuprofen, IBU, and triclosan, TCS) and their mixtures (IBU+TCS) at different time exposure. Period 0 (control); Period I (7 days) and Period II (14 days).

The results from fluorescence microscopy are depicted in Fig 4, presenting the viability status of AS bacterial cells before and after exposure to IBU, TCS and IBU+TCS, respectively. During Period I no significant effect was observed maintaining a large majority of viable cells, whereas in Period II the bacteria presented a clear membrane integrity decline. The non-reversible damage on AS viability was associated to the increase of oxidative stress and antioxidant enzymes levels.

## 4. Conclusions

Performance, oxygen respiration rate, viability and microbial enzymatic activity were investigated in AS reactors continuously exposed to IBU and TCS, alone and in combination, for 14 days, and the results related to the oxidative stress suffered by bacterial cells. IBU had no significant impact on reactor performance, while TCS led to significant decrease of COD removal efficiency. Toxic effects occurred upon continuous long-term dosages of the selected PPCPs. The toxicity to AS was caused by ROS generation and membrane integrity decline. Activity of key enzymes was affected by long-term exposure to IBU and TCS. Overall, our results showed for the first time that AS bacteria have the capacity to tolerate oxidative stress by activating their antioxidant system under continuous dosage of IBU and TCS.

## Supporting information

S1 File Variations in effluent performance of single (ibuprofen, IBU, and triclosan, TCS) and mixed (IBU+TCS) reactors during continuous feeding operation (Figure A. O_2_ dissolved; Figure B. pH; Figure C. Biomass conc.). (DOCX)
