# Supplementary figures and images for: Enzyme response of activated sludge to a mixture of emerging contaminants in continuous exposure
Source: PLoS One. 2020 Jan 13;15(1):e0227267. doi: 10.1371/journal.pone.0227267 (PMC6957336; doi:10.1371/journal.pone.0227267)

**
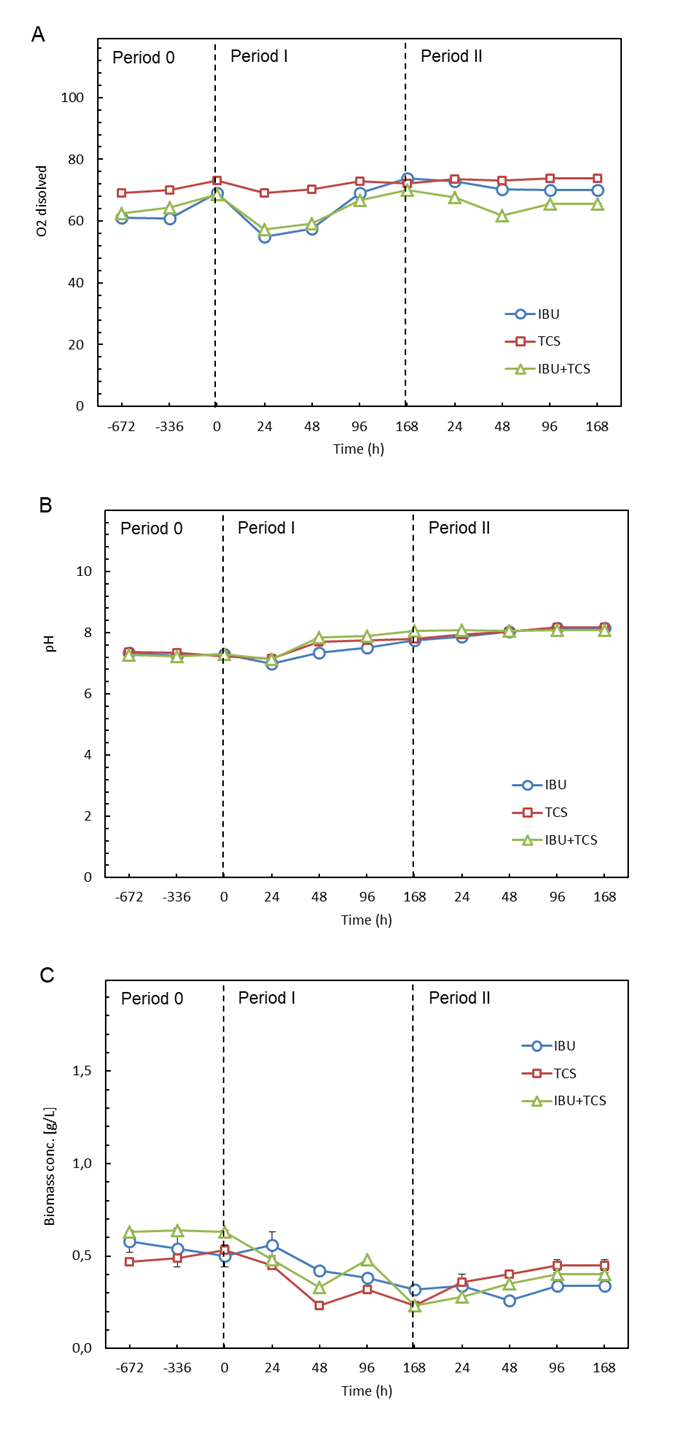
**

Supplement: S1 File — (DOCX) [file pone.0227267.s001.docx]
